# Historical Researches on Pemphigus

**Published:** 1814-10

**Authors:** M. Savary


### *For the Medical and Physical Journal.*

### *Historical Researches on Pemphigus; by* M. SAVARY.

M. GILIBERT, in the excellent Treatise he has given on Pemphigus, has passed very lightly over the history of this disease. The following remarks, which were began before the publication of his work, may possibly have some interest. We have, therefore, thought it adviseable to present them to the public.

The term Pemphigus being derived from the Greek *πιμφιξ* or *πεμφιγξ*, it is proper to revert to the writers in that language for its meaning. Hippocrates speaks of a fever called Pemphigodes, but what he has said respecting it is insufficient to characterize it. His expressions are, *οι δε πεμφιγοδης ιδειν δεινοι*,* *aliæ pemphigodes aspectu terribiles*—the word *πυρετοι*, *febris*, is understood.

The long commentaries of Galen on this passage have only rendered it still more obscure. He speaks of variations in the manuscript copies, some containing the two first words only, others with the text as above. He then affixes different significations to the term; sometimes he believes it to indicate an exhalation from the skin of the patient, at others he supposes the febris pemphigodes to be those which are accompanied by pustules. Again, he will have it a pestilential fever analogous to that described by Thucydides, or, taking the word *πεμφιξ* in a figurative sense, to signify soul or mind. He says the fever is characterised by an affection of the intellectual functions, or delirium. We shall, however, leave all this interpretation, and keep to the definition of this fever, which is to be found in another work ascribed to the same author.† *The febris pemphigodes is that which*

---

* De Morb. vulg. lib. vii. Fæs.

† Finitiones Medicæ in Isag. Fol. 46.

*generates pustules in the mouth, called by the Greeks Phlyctides.*

But supposing, what is very possible, that the *febris pemphigodes of Hippocrates* was not the *pemphigus of the moderns*, it is evident the latter was not unknown to the father of medicine, which the following passage from the 2d Book of Epidemics sufficiently proves. "Ichores quidem cuti subnascebantur qui intro concepti calescebant, pruritum que concitabant deinde phlyctænides ambustis pustulis similes, assurgebant quibus sub cutem uri videbantur."

The same passage teaches us what the Greeks understood by Φλυκτενειδες, and consequently by Φλυκτιδες and Φλυκταιναι: all these are in fact synonymous terms, and signify little bladders, filled with serosity, like those which arise in consequence of a burn. The most proper place, therefore, to obtain information respecting the disease, is from what the ancients have said of phlyctenæ. Celsus, in speaking of pustules, called Φλυκταιναι ελκωδεις, says, with his accustomed brevity, "ubi hæ ruptæ sunt infra quasi exulcerata caro apparet. Fiunt vel a frigore vel ex igni vel ex medicamentis."* Ætius expresses himself more clearly:† "Papulæ quibusdam oriuntur similes his quæ a fervida aqua ambustis emergunt non tamen multum dolorem inducentes. Quibus ruptis subflavi humoris copia paulatim effluit ad biduum aliquando ac triduum durans." The vesicles of pemphigus are here perfectly described, but nothing is said of the fever which commonly accompanies and always precedes them. Ætius adds, that this eruption is more common in women who menstruate irregularly, and in children.

The disease described by Rhases under the name of *Ignis sacer*,‡ seems analogous to the phlyctenæ and pemphigus. He speaks of it as an exanthema, which consists in bladders like those of a burn, and says, that they are commonly preceded by redness and itching, and terminate by ulcers covered with a dark crust. He then adds, that the fever which accompanies this eruption is acute, and insidious in its course. All these symptoms are characteristic of the pemphigus, accompanied with typhus fever.

Fernel,§ who arranges phlyctenæ under erisipelas, gives a description of them very similar to that of Ætius. These are his expressions: "Repente emergunt subflavi humoris copia distentæ, bullarum modo lucidæ atque iis similes quæ

* De re Medica, lib. v. cap. 28.

† Tetrab. iv. serm. 11. cap. 63.

‡ Lib. 27, tract 7, tom. 2.

§ Univers. Med. lib. vii. cap. 4.

ab aqua fervidâ vel ab igne ambûstis erumpunt non manifesto tamen dolore graves his ruptis humor excidit crustulæque obdurescunt dum sanescunt."

Rondelet,* Nicholas Lepois,† Sennertus,‡ Felix Plater,§ and Musitanus,|| have copied Ætius and Fernelius in what they have said of phlyctenæ. Forestus describes them in the same terms, and relates a particular case, which we shall transcribe, as it appears to be the first upon record. It is entitled "de Phlyctænes in facîe cujusdam infantis apparentibus."¶ "A child had transparent vesicles on the face, filled with a yellowish fluid, similar to those which follow a burn produced by boiling water or fire; but they were not attended with much pain. The vesicles broke, and poured out their fluid, after which they were covered with crusts. While healing, I advised the pustules to be anointed first with the juices of plaintain and nightshade, then with a mixture of chalk and vinegar, under the form of a liniment. I washed the face also with a decoction of roses, and prescribed to the nurse cooling diet, with verjuice, and skimmed milk for drink; and in a short time she recovered."

Another case, published at the same time by Schenkius,** deserves also to be mentioned, as it shows that pemphigus was observed before the end of the sixth century. The subject of the observation was the son of this physician. In the space of two days his head, and particularly the face, swelled in an extraordinary manner, and there appeared on the chest, back, and arms, oblong pustules, or rather, says the reporter, transparent vesicles, as though they had been occasioned by the affusion of boiling water, or stinging with nettles, accompanied with itching and redness of the face. After a complicated treatment, which it is not necessary to relate, the disease quickly disappeared—tumor omnis brevi evanuit.

In 1618, Charles Lepois gave a case, in which pemphigus is easily recognised, though the disease is designated hydatides.†† M. Gilibert has referred to it in his work as a complication of pemphigus with putrid fever.‡‡ The same au-

---

* Method. Curand. Morb. cap. 50.

† De cog proscendio & Curandis morbis, lib. i. cap. 32.

‡ Med. Pract. lib. v. De tumorib. cap. 22.

§ Prac. Med. de superf. corp. dolore, cap. 17.

|| Trutina Chirurgico-Physica, de humor, cap. 25.

¶ Obs. Chirurg. lib. ii. cap. 8. 1590.

** Obs. Medicæ raræ, lib. v. obs. 124.

†† Select. Obs. & Consil. obs. 149.

‡‡ Monographie du Pemphigus, page 160.

thor thinks,* with Sauvages, that the epidemic disease which prevailed in India in the year 1628, and is described by Bontius,† belongs to pemphigus, or is at least complicated with it; but this appears doubtful, the appearances mentioned by Bontius being insufficient to characterise the eruption. He merely says, that, in the patient whose case he relates, *pustules and vesicles, filled with greenish pus*, appeared, which corroded the skin in the axillæ and groins, upon the neck, back, and loins. Nothing is said of *transparent vesicles and limpid fluid.* Sauvages indeed adds, "Observavit etiam in amphemerina epidemica ab anno 1658 ad 1691. Vesiculas aqueas per collum & pectus sparsas Bontius."‡ But, besides that this description is not to be found in the works of Bontius, this author being dead in 1631, there is in the assertion of Sauvages an evident error. Zacutus Lusitanus is referred to as having observed pemphigus. This physician, it is true, relates a case under the title of Febris pemphigodes, febris ampullosa seu Bullosa;§ but the observation is so lame, that no conclusion whatever could have been drawn from it, if the author had not himself defined the Febris ampullosa, which proves that it is not the true vesicular disease. "I thus call a fever," says he, "in which tubercles appear on the body." A little farther, he says, "that tubercles were by the ancients denominated vibices;" and, in the observation which follows, speaks of pustules. How shall we find pemphigus in tubercles, vibices, and pustules?

We shall be equally deceived if we place to this account the observations collected by Riedlin. One is entitled *Pemphinx retrocessens animi deliquium causatur;*|| and the other *Pemphinx or essere.*¶ In the first he is speaking of eminences or protuberances on the surface of the skin, like the bites of purmises, or the stings of cousins; in the second he speaks of pustules whose eruption has been preceded by intolerable itching.

The eruption described by Sydenham in the following passage, has more analogy with pemphigus; but this is merely accidental or symptomatic: "Pustularium per universum fere corpus eruptio, quæ urticarum puncturas referunt & nonnunquam in vesiculas attolluntur."**

---

* Monographie du Pemphigus, p. 173.

† De Medicina Indorum Obs. Select.

‡ Nosol. Method. tom. i. p. 432. Edit. Amst. 1768, in 4to.

§ Prax. Med. Adm. lib. iii. obs. 15.

|| Obs. Med. cent. i. obs. 23. ¶ Ibid. cent. iii. obs. 48.

** Obs. Med. circa morb. acut. sect. vi. cap. 6. de Febris erysipelatosa.

Morton

Morton also includes this eruption with those which appear in malignant fevers.

A singular fact is related by Simon Schulz,* which we really think belongs to pemphigus. A woman, thirty-eight years of age, regularly, for some days previous to the menstrual discharge, suffered from vertigo and pain in the head, which was soon followed by the eruption of a vesicle behind each ear. These vesicles ruptured spontaneously, pouring out a humour of a yellowish colour; and immediately after the menses appeared. This observation was communicated to the society *Naturæ Curiosorum* in 1676. In the year following, Christopher Seliger presented his case,† which M. Gilibert places with his first species. The same collection contains many others, of which we shall speak hereafter. In 1691, Paul Spindler gave a case in which it is easy to discover the character of pemphigus.‡ "A young person, after cold chills, succeeded by heat, had upon the abdomen an eruption of vesicles, preceded by an appearance of flea-bites. These vesicles were filled with serum, and occasioned a lancinating pain. The appetite was lost; the pulse was stronger, and more frequent than natural; the urine high-coloured." The author observed the same complaint in several individuals that year. Hoffman considers this case to be analogous to one which he published.§ Other facts relating to pemphigus are mentioned by the latter in the Consultat et respons, cap. 123.||

The following observation by Lamotte presents to us a case of erisipelas complicated with pemphigus. It is, in point of date, much anterior to those furnished by Delabrousse.

"In March, 1698, a magistrate of our town was suddenly attacked with cold chills, which continued upwards of two hours, and were followed by violent fever, and a distressing itching in the face, with heat in the eyes, and a continual flow of tears. When the fever was a little diminished, I bled him. His face after this became swoln and red, the bleeding was repeated, a glyster administered, and linen moistened with brandy was applied to the part. During the night the face became covered with phlyctenæ, and on the following day I found him apparently as if boiling water had been poured upon him. The brandy having produced much

* Eph. Nat. Cur. dec. i. ann. vi. et. vii. obs. 233.

† Ibid. dec. i. ann. viii. obs. 56.

‡ Obs. Med. Franc. 1691, in 4to. obs. 92.

§ De affectu raro Scorbutico.

|| Also in Med. Ration. part v. cap. 5, under the title of *Affectus pustularis pruriginosius.*

pain, pain, I was obliged to remove it, and substitute in its place soft cream, which I applied as a liniment to the face, ears, and that part of the throat, which was occupied by the disease, the patient soon recovered."*

Following a chronological order, we ought to speak here of the treatise of J. J. Fiek, on the vesicular fever caused by the retention of the lochia;† but we know nothing of this book except the title; and perhaps this pretended vesicular fever is nothing but the miliary fever of child-bed women. After this came the observations of Delius,‡ Furztenau,§ Freutzel,‖ the descriptions of Thierry,¶ and of Langhans,** from which Sauvages has established several species. This author distinguishes, in fact, five species of pemphigus.†† The first he calls Pemphigus Major, from the observations of Seliger, Lepois, and Delius. The second, Pemphigus Castrensis; it is that which Thierry has rather pointed out than described. The third, described by Langhans, the Pemphigus Helveticus. The fourth, Pemphigus Indicus, from Bontius and Morton. Lastly, he makes a species of the vesicular eruption of Bougeant, and he denominates it Pemphigus Brasiliensis.‡‡

Linnæus, whose classification of diseases is of the same date as that of Sauvages, only speaks of the Symptomatic Pemphigus, under the name of *Morta*. Vogel calls it Febris Bullosæ,§§ but never saw it. Macbride, who never met with it in his practice, calls it Febris Vesicatoria; but he describes this fever as having some analogy with the plague, and the Chronic Pemphigus is ranged under the class Cachexiæ; nevertheless, these two eruptions, as Mr. Gilibert has proved, do not differ essentially. The distinction made by Daniel,‖‖ between the external or Common Pemphigus, and the internal or Helvetick Pemphigus, is equally unfounded. Sagar

---

* Traité complet de Chirurgiæ.

† De Febre vesciculari ab abstractione Lochionum, Jena, 1726, in 4to.

‡ Amænit. Med. Dec. v. cap. ix. Febris Vesicular.

§ Act. Nat. Cur. tom. ix. Obs. 18. Febris Acuta Vesiculoso Erisipelacea.

‖ Ibid. tom. x. p. 260. De peculiari quadam febris malignæ. Catarrhalis exanthematicæ, purpuraceo pustularis specie, vesicali ulcerosa.

¶ Med. Experim. part i. chap. v. sect. 3.

** Act. Helvet. tom. ii. p. 260. Brevis delineatio morbi qui anno 1752, in Valle Simmia epidemice grassatus est.

†† Nosolog. Method. 1768, tom. i. p. 430, 4to.

‡‡ Observ. sur la Physique, tom. i.

§§ De Cognoscendis & Curandis morbis.

‖‖ Syst. Ægritud. Lips. 1781, in 8vo. p. 147.

has has added to the species of Sauvages the Pemphigus Apyreticus;* whilst Plenck† has reduced them to four. Cullen contented himself with enumerating the species of Sauvages, with doubts respecting the existence of the disease, which at that time he had not seen, though Dr. Home afterwards pointed it out to him.

Among the particular observations, we ought not to pass by a well-established fact related by Van Swieten.‡ A man, 50 years of age, of a good constitution, in the habit of taking largely of wine, began, on the 27th of July, to experience pain in the back, and gradually, without any febrile affection, this part, for the breadth of three inches, was covered with little pustules depressed in the centre, containing a yellowish humour; they became confluent in many places, and on the first of the ensuing month the subjacent parts appeared black; the urine was red, voided in small quantity, with a white sediment; the appetite was lost, without much thirst; the tongue was clean, with no bad taste in the mouth. On the 16th of the month he was quite cured.

Another distinguished practitioner, Rougnon, of Montpellier, saw it reign epidemically at a time when bilious complaints were prevalent.§ M. Sourtelle also observed it at Besançon at the same time.|| Finke met with a very remarkable instance in an epidemic bilious fever.¶ Dickson,** and after him Miroglio,†† have seen the disease in all its simplicity; and Salabert, in a constitution eminently bilious, has seen the vesicular eruption manifest itself in a critical manner.‡‡ Burghard had before related an instance of the same kind.§§ Lastly, Blagden has related two cases which deserve particular attention, inasmuch as they seem to indicate a contagious property in the disease not allowed by others.||||

"I was called," says Blagden, "in January, 1790, to see a little girl; she had fever; I gave an emetic; the following day the fever was stronger, and she had delirium; a blister was applied between the shoulders, and the bowels were opened by an emollient injection. In the evening pustules

* Syst. Morb. Sympt.

† Doct. de Morb. Cutan. Cl. iv. gen. 3. Pemphigus.

‡ Constit. Epidem. published by Stoll, after his death. Stoll, tom. i. p. 125.

§ Considerat. Pathologicæ Semeioticæ, fasc. i. p. 231.

|| Elemens de Med. tom. ii. p. 126.

¶ De Morbis Bilios. anom. p. 118.

** Journale de Med. tom. 80, p. 178.

†† Ibid. tom. 81, p. 273. ‡‡ Ibid. tom. 82, p. 72.

§§ Nov. Act. Nat. sur tom. iii. p. 328, Obs. 72.

|||| Medical Facts, tom. i. p. 105.

appeared, appeared, first on the chest, and afterwards on the hairy scalp; and other parts of the body, in three days; they filled with a yellowish fluid, and had the appearance of little bladders; the greater part were of the size of an almond, but those of the forehead and chest were as large as a walnut; the largest were opened, the others suffered to break of themselves. Cerate was used for dressing the vesicles of the limbs, and those of the head were cured in about ten days, but those of the chest were exquisitely painful, and lasted two months; some on the forehead left marks behind, which were never afterwards effaced; small vesicles were observed in the mouth, and the child shewed great reluctance in swallowing, as the fever and delirium disappeared immediately after the eruption; the disease was trusted to nature, no pustule remained after the fourth day."

Five days after the appearance of this eruption on the first child, another, three months and a half old, belonging to the same parents, was seized with fever, and in the course of the three following days had an eruption exactly similar, except that the vesicles did not exceed the size of a pea. They ruptured spontaneously, and the little wounds cicatrized in the space of a week. This child required no medicine.

Wichman made this disease the subject of a particular dissertation,* where he relates several cases of Chronic Pemphigus. J. P. Franck gave an extensive article of it in his Epitome.† Reil speaks of it at length.‡ Burserius describes it very well.§ It has been also described by Willan,‖ Thomas,¶ Darwin,** Pinel,†† and M. Swediaur.‡‡ We pass by the recent observations published in the different journals of medicine, because they are all found, and for the most part analysed in Mr. Gilibert's work. But there are three dissertations with which he appears to be unacquainted: the first is that of Mr. Charles Bobba; §§ the second, by Mr. Eckhout;‖‖ the last, by M. Bunel. ¶¶ The memoir of M. Robert may be ranged with these.***

---

* Beytrag zur kentniss Pemphigus, Erf. 1790, in 4to. de 16 page.

† De Curand. Homin. Morb. tom. ii. p. 363.

‡ Memorab. Clin. Fasc. II. sect. vi. p. 145.

§ Instit. Med. Pract. tom. ii. p. 92.

‖ On Cutaneous Diseases.

¶ Practice of Physic.

** Zoonomia, tom. iii. p. 404.

†† Nosograph. Philosoph. tom. ii. p. 91, 3d edit.

‡‡ Nov. Med. rat. systema. tom. i. p. 166.

§§ Memoire sur le Pemphigus ou exantheme vesiculare. Stuttg. 1802, in 8vo.

‖‖ De Pemphigo. Groning. 1810, in 8vo. Diss. Inaug.

¶¶ Essai sur le Pemphigus. Paris, 1811, in 4to. Diss. Inaug.

*** Journ. de Med. continué, tom. xxiv. p. 3, 107, 219, et 327.

